# Newly diagnosed incident dizziness of older patients: a follow-up study in primary care

**DOI:** 10.1186/1471-2296-12-58

**Published:** 2011-06-24

**Authors:** Julia Sczepanek, Birgitt Wiese, Eva Hummers-Pradier, Carsten Kruschinski

**Affiliations:** 1 Institute of General Practice, Hannover Medical School, Hannover, Germany; 2 Centre for Biometry, Medical Informatics and Medical Technology, Hannover Medical School, Hannover, Germany

## Abstract

**Background:**

Dizziness is a common complaint of older patients in primary care, yet not much is known about the course of incident dizziness. The aim of the study was to follow-up symptoms, subjective impairments and needs of older patients (≥65) with incident dizziness and to determine predictors of chronic dizziness. Furthermore, we analysed general practitioners' (GPs') initial diagnoses, referrals and revised diagnoses after six months.

**Methods:**

An observational study was performed in 21 primary care practices in Germany, including a four-week and six-month follow-up. A questionnaire comprising characteristic matters of dizziness and a series of validated instruments was completed by 66 participants during enrolment and follow-up (after 1 month and 6 months). After six months, chart reviews and face-to-face interviews were also performed with the GPs.

**Results:**

Mean scores of dizziness handicap, depression and quality of life were not or only slightly affected, and did not deteriorate during follow-up; however, 24 patients (34.8%) showed a moderate or severe dizziness handicap, and 43 (62.3%) showed a certain disability in terms of quality of life at the time of enrolment. In multivariate analysis, n = 44 patients suffering from chronic dizziness (dependent variable, i.e. relapsing or persistent at six months) initially had a greater dizziness handicap (OR 1.42, 95%CI 1.05-1.47) than patients with transient dizziness. GPs referred 47.8% of the patients to specialists who detected two additional cases of benign paroxysmal positional vertigo (BPPV).

**Conclusions:**

New-onset dizziness relapsed or persisted in a considerable number of patients within six months. This was difficult to predict due to the patients' heterogeneous complaints and characteristics. Symptom persistence does not seem to be associated with deterioration of the psychological status in older primary care patients. Management strategies should routinely consider BPPV as differential diagnosis.

## Background

Dizziness is a frequent complaint of older patients in primary care. Its point prevalence increases with age up to a total of 30% of people 65 years of age and older [1-4]. For the general practitioner (GP) two aims are crucial: to exclude a life-threatening or treatable specific disease and to identify a chronic development of dizziness. As to exclusion of life-threatening diseases, Bird et al. [5] found that GPs did not fail to refer the rare urgent cases. Altogether, more than one in six patients were referred inappropriately, which was more common in older people. In other studies, serious causes of dizziness were not overlooked [6-8]. However, these studies did not concentrate on the specific situation of older patients.

The diversity of possible aetiologies concerning multiple organ systems and associated risk factors yielded the hypothesis that dizziness represents a chronic multifactorial geriatric syndrome [4,9,10]. For many older patients especially the deterioration of spatial perception and its concomitant features produce a debilitating experience impairing health-related quality of life [11]. Accordingly, studies revealed associations between anxiety, depressive and somatoform disorders as well as restrictions in daily life and social activities in chronic dizziness [3,11-13].

A considerable number of early studies conclude with the requirement for prospective data, as little is known about the dizziness-associated alterations of patient characteristics (e.g. dizziness handicap, patients' needs) in relation to the time-dependent course of the symptom [14-16]. Considering this together with the two above-mentioned aspects that the GP is mostly concerned about, we initially aimed to investigate the development of incident dizziness, the associated patients' impairments and needs, and to assess predictors of chronic dizziness. In parallel, we wanted to analyse GPs' preliminary diagnoses, referrals and possible work-up diagnoses.

## Methods

### Design of the study and enrolment

The study design was a prospective observational follow-up study. Participants were consecutively registered in 21 primary care practices in the city and region of Hannover, Germany. Inclusion criteria were an age of at least 65 years and incident dizziness (which had been present for less than six months and not yet shown to another doctor) as main reason for the encounter. Exclusion criteria comprised insufficient command of the German language, dementia, or terminal diseases.

All participants gave informed consent. The study was approved by the Ethical Committee of Hannover Medical School (number 4291).

### Questionnaire design: Assessment of patients' impairments and needs

At the time of enrolment, patients filled in a standardised questionnaire containing sociodemographic parameters and a detailed description of the dizziness symptoms. This included the duration, the classification according to Drachman and Hart [17] (presyncope, vertigo, disequilibrium or lightheadedness/other), triggers and concomitant symptoms. The Dizziness Handicap Inventory (DHI, [18]) was used to assess the degree of disability associated with any cause of dizziness. The Dizziness Needs Assessement (DiNA, [19]) was used to assess wishes and needs of the patients concerning their dizziness complaints. Anxiety was assessed using the following question (complying with an established screening instrument for anxiety [20]; dichotomous response: yes/no): "In the past four weeks, have you been compromised by anxiety or a feeling of being emotionally out of balance?" The Geriatric Depression Scale (GDS, [21]) was applied as a basic screening for depression in older people, and the 12-item Short Form Health Survey (SF-12, [22]) was used to analyse quality of life. Activities of Daily Living (ADL, [23]) were assessed, including basic abilities such as eating and drinking, and more complex instrumental abilities (iADL) such as doing the housework. Some of the instruments were slightly modified or culturally adapted according to the specific circumstances in Germany. The explanation of the resulting scores is displayed in the table legends.

Participants filled in a questionnaire containing the same instruments one month and six months after the first consultation. Moreover, the follow-up questionnaires contained information about the persistence and intensity of the symptom. Patients were phoned or visited in order to complete missing data.

### Chart Review: Assessment of the doctors' diagnoses

After six months, each of the surgeries were visited in order to perform a chart review of all patients by interviewing each GP. The ICD (International Classification of Diseases)-10-code or "free" documentation of how the GP had initially diagnosed the dizziness was specified in a face-to-face interview. Here, GPs expressed their assumptions of the aetiology of dizziness. Information on possible causes of dizziness was actively requested according to a predefined check-list. Moreover, referrals to specialists as well as the corresponding follow-up diagnoses were documented.

### Statistical Analysis

All statistical analyses were performed using the SPSS statistics software (version 17.0). For analysis of the development of the patients' impairments and needs over time, the non-parametric Friedman test was used to explore differences of test scores and Likert scale items (DiNA, [19]).

For further analysis, all patients were divided into two groups: temporary dizziness (present only at the time of inclusion or persisting at the four-week follow-up but not at six months), and chronic dizziness, i.e. relapsing (not present at the four-week follow-up, but recurring at the six-month follow-up) or persistent (dizziness present at all three time points of investigation). To explore differences of test score results and Likert-scale items between the temporary and chronic dizziness groups at each time point, the Mann-Whitney-U-Test was used.

Multivariate logistic regression analysis was used to identify predictors of chronic dizziness. For inclusion in the model, variables were selected on the basis of significance taking into account the clinical relevance of the differences and the literature. For example, "depression" is a relevant co-morbidity in chronic dizziness [13] and was therefore included. Univariate and multivariate Odds Ratios (OR) with their 95% confidence intervals (CI) were calculated for all variables.

For all statistical analyses, variables were considered significant if p < 0.05.

## Results

### Sociodemographic characteristics

We prospectively identified 77 patients presenting at their GPs' with a chief complaint of incident dizziness. Eight patients did not fulfil the inclusion criteria due to age (1 patient) or the duration of dizziness (7 patients). Three patients were not followed up and thus completed questionnaires from all three time points were obtained for 66 patients. The mean age of all 69 participating patients with dizziness was 76.19 years (SD = 6.64 years, range 65-95). The majority of patients were female (n = 48, 69.6%). The characteristics of dizziness are shown in table 1.

**Table 1:** Characteristics of dizziness.

	n	%
Type of dizziness*		
unsteadiness	45	65.2
vertigo	32	46.4
fainting	11	15.9
other e.g.	12	19.4
feeling of swaying from one side to the other	38	55.9
lift feeling	11	15.9
		
**Duration**		
seconds	25	41.0
minutes to hours	19	31.1
several hours	10	16.4
continuous	7	11.5
		
Dizziness eliciting situations*†		
getting up from lying or sitting	39	56.5
bending down	36	52.2
head turning	23	33.3
rotatory movement	21	30.4
lying down	19	27.5
walking	17	24.6
standing	13	18.8
walking on uneven ground	13	18.8
darkness	11	15.9
lying on one side	9	13.0
		
Concomitant symptoms*†	46	66.7
nausea	24	34.8
tinnitus	18	26.1
headache	16	23.2
tachycardia	12	17.4
weakness (extremities)	9	13.0
muscle tremor	8	11.6
vomiting	8	11.6
dyspnoea	7	10.1
numbness (extremities)	7	10.1
perspiration	7	10.1
visual disturbance	7	10.1

* more than one answer possible

† symptoms listed only if present in more than 10% of cases

### Psychological/functional impact and patients' needs in terms of dizziness during follow-up

The course of dizziness and its associated implications were investigated over the 6-month period (table 2). Most mean instrument scores did not indicate pathological values at enrolment (T0), except a moderate restriction in terms of quality of life (SF-12). However at T0, the proportion of "disabled" patients (SF-12, score less than 51) was 43/69 (62.3%), and the proportion of patients showing a moderate or severe dizziness handicap (modified DHI, score 26 or more) was 24/69 (34.8%).

**Table 2:** Development of the different instrument scores during follow-up (mean ± SD).

Instrument	Explanation/Item	T0	T1	T2	P*
**DHI **total†	Dizziness handicap	26.68 ± 13.27	22.95 ± 15.12	24.32 ± 15.24	0.055
physical	(subscale)	7.05 ± 3.59	6.00 ± 3.97	6.21 ± 3.91	0.195
functional	(subscale)	11.42 ± 7.41	10.68 ± 8.44	11.57 ± 8.55	0.164
emotional	(subscale)	8.21 ± 5.44	6.26 ± 6.10	6.53 ± 5.97	0.036
**SF12**	Quality of life	47.03 ± 11.61	49.41 ± 10.08	48.64 ± 9.94	0.639
**GDS**	Depression	3.19 ± 2.90	3.08 ± 2.94	2.63 ± 2.76	0.174
**ADL **total	Activities of daily living	39.63 ± 4.05	39.72 ± 4.08	39.53 ± 3.29	0.407
basic	(subscale)	15.23 ± 1.47	15.11 ± 1.62	15.28 ± 1.13	0.620
instrumental	(subscale, e.g. financial tasks)	20.67 ± 2.52	20.84 ± 2.30	20.56 ± 2.15	0.491
					
**DiNA**	Importance of knowing the cause	4.91 ± 1.42	4.82 ± 1.38	4.91 ± 1.18	0.465
	Risk of falling	3.47 ± 1.52	3.09 ± 1.42	3.15 ± 1.42	0.300
	Effectiveness of self-help measures	3.80 ± 1.47	4.30 ± 1.42	4.15 ± 1.35	0.368
	Handicap during acute dizziness	4.03 ± 1.52	3.76 ± 1.55	3.49 ± 1.57	0.058
	General dizziness handicap	3.03 ± 1.42	2.72 ± 1.34	2.67 ± 1.35	0.028
	Empathy of doctor	5.22 ± 1.20	4.69 ± 1.37	4.25 ± 1.52	< 0.001
	Help of doctor	2.83 ± 1.39	3.07 ± 1.60	2.87 ± 1.50	0.630

T0, T1, T2 represent the three time points of assessment (see legend).

T0 first time point of assessment

T1 second time point of assessment (four-week follow-up)

T2 third time point of assessment (six-month follow-up)

* Differences of mean test score and Likert scale items (DiNA) comparing the three time points were tested using the Friedman test

† scores: DHI/modified (maximum score [= highest handicap]: 88, ≥27: moderate or severe handicap; adapted according to [31]), SF-12 (≥51: "no disability", ≤30: "severe disability"), GDS (< 6: "no depression", > 6 and < 11: "mild to moderate", ≥11: "severe"), ADL/modified (maximum score [= lowest handicap]: 42 [instrumental (iADL): 22, ADL: 16, continence: 4]), DiNA (Likert scales 1-6 [growing with increasing numbers])

The mean scores of most instruments were relatively stable over time. However, in terms of the dizziness handicap there was a difference depending on the instrument used. The DiNA (patients' needs), in which the "handicap during acute episodes of dizziness" can be differentiated from a "general dizziness handicap", revealed that the handicap during acute dizziness was high at the beginning and decreased significantly over time. This was different from the "general dizziness handicap" (DiNA) as well as from the handicap as measured by the DHI.

Patients considered it very important to know the cause of the dizziness (DiNA), which remained stable over time, and to be taken seriously by their doctor ("empathy of doctor"), though this aspect decreasing significantly over time. Anxiety screening was positive in 41.2% of patients at the time point of first assessment.

### Comparing patients suffering from temporary or chronic dizziness

Apart from the intraindividual follow-up of the patients' impairments and needs (table 2), patients were analysed separately according to whether they belonged to the temporary (n = 22) or chronic dizziness group (i.e. the dizziness persisted after the 6 months follow-up, n = 44), at each time point of assessment (table 3). Although neither group achieved true "pathological" mean values, patients suffering from chronic dizziness manifested stronger impairments in terms of dizziness handicap (DHI) and ADL than those of the temporary group at the time of enrolment as well as during follow-up (ADL). Considering the patients' needs (DiNA), the perceived GP's capability of helping with their dizziness ("help of doctor") was rated significantly higher in the temporary group in comparison to the chronic group at the time of first assessment.

**Table 3:** Instrument scores (mean ± SD) comparing temporarily dizzy (not any more present after six months, n = 22) and chronically dizzy (still present after six months, n = 44) patients at the three time points of investigation.

Instrument	Explanation/Item	T0 temporary	T0 chronic	P*	T1 temporary	T1 chronic	P*	T2 temporary	T2 chronic	P*
**DHI**	**Dizziness handicap**	15.27 ± 14.42	25.36 ± 13.90	**0.004**	18.80 ± 17.64	22.95 ± 15.12	**0.258**	n.a.	22.59 ± 15.14	**n.a**.
**SF12**	**Quality of life**	49.09 ± 11.63	45.82 ± 11.34	**0.294**	51.76 ± 9.75	48.5 ± 10.16	**0.279**	51.4 ± 7.63	47.39 ± 10.67	**0.178**
**GDS**	**Depression**	2.41 ± 2.15	3.66 ± 3.14	**0.138**	2.52 ± 2.82	3.32 ± 2.96	**0.265**	2.10 ± 2.27	2.86 ± 2.95	**0.287**
**ADL**	**Activities of daily living**	41.14 ± 2.17	38.95 ± 4.50	**0.001**	40.86 ± 2.33	39.18 ± 4.58	**0.013**	40.67 ± 2.22	39.05 ± 3.58	**0.045**
										
**DiNA**	**Importance of knowing the cause**	5.18 ± 1.44	5.12 ± 1.31	**0.769**	5.20 ± 1.32	4.67 ± 1.41	**0.309**	n.a.	5.02 ± 1.18	**n.a**.
	**Risk of falling**	3.00 ± 1.61	3.37 ± 1.57	**0.317**	2.80 ± 1.87	3.08 ± 1.38	**0.356**	n.a.	3.07 ± 1.57	**n.a**.
	**Effectiveness of self-help measures**	4.07 ± 1.39	3.52 ± 1.60	**0.268**	4.25 ± 1.83	4.11 ± 1.45	**0.659**	n.a.	3.75 ± 1.72	**n.a**.
	**Handicap during acute dizziness**	4.29 ± 1.55	3.98 ± 1.58	**0.392**	3.70 ± 1.34	3.76 ± 1.55	**0.905**	n.a.	3.39 ± 1.56	**n.a**.
	**General dizziness handicap**	2.76 ± 1.55	3.09 ± 1.49	**0.360**	2.40 ± 0.97	2.72 ± 1.34	**0.471**	n.a.	2.52 ± 1.34	**n.a**.
	**Empathy of doctor**	5.62 ± 0.59	5.32 ± 1.14	**0.552**	5.30 ± 1.06	4.69 ± 1.37	**0.180**	n.a.	4.27 ± 1.58	**n.a**.
	**Help of doctor**	4.26 ± 1.56	2.93 ± 1.46	**0.004**	3.33 ± 1.80	3.13 ± 1.61	**0.729**	n.a.	3.07 ± 1.59	**n.a**.

Scores are explained in the legend of table 2.

T0 first time point of assessment

T1 second time point of assessment (four-week follow-up)

T2 third time point of assessment (six-month follow-up)

* Differences of mean scores and Likert-scale items between the temporary and chronic group at each time point were tested using the Mann-Whitney-U-Test

n.a. not applicable

Moreover, patients' responses were evaluated using logistic regression analysis in order to define predictors of chronic dizziness. For this purpose, "chronic dizziness" (yes/no) was defined as the dependent variable (outcome). For the covariates included (see table 4) only the scores of the time point of inclusion (T0) were considered, as we were interested in whether these variables could help the GP at the first time of encounter to anticipate whether dizziness would become chronic. In this multivariate analysis, a greater handicap, as measured by means of the DHI, was associated with chronic dizziness. A patient's perception that the GP would be unable to help with respect to their dizziness ("How much can your doctor help you in terms of your dizziness?") at T0, was also associated with chronic dizziness, and a positive anxiety screening was negatively associated with chronic dizziness.

**Table 4:** Predictors for chronic dizziness including univariate and adjusted OR and 95% CI.

	univariate analysis			multivariate analysis		
	**OR**	**95% CI**	**P**	**OR**	**95% CI**	**P**

age	1.07	0.97-1.16	0.196	1.09	0.91-1.32	0.352
female gender	0.87	0.25-3.03	0.824	0.51	0.04-6.02	0.591
**Type of dizziness**						
vertigo	0.48	0.15-1.53	0.214	1.27	0.12-14.05	0.846
fainting	5.93	0.69-50.70	0.104	13.78	0.41-465.80	0.144
unsteadiness	1.47	0.43-5.05	0.538	0.24	0.01-4.92	0.355
other form	0.76	0.19-3.05	0.697	0.12	0.01-2.51	0.170
**Instruments**						
GDS*-Score	1.27	0.99-1.62	0.061	1.55	0.80-3.00	0.190
anxiety (screening)	2.98	0.91-9.74	0.071	0.01	0.00-0.43	0.016
DHI†-Score	1.12	1.04-1.20	0.002	1.24	1.05-1.47	0.013
help of doctor (DiNA‡)	0.54	0.35-0.84	0.003	0.37	0.15-0.94	0.037

* Geriatric Depression Scale

† Dizziness Handicap Inventory

‡ Dizziness Needs Assessment

### GPs' diagnoses and referrals

In parallel to the description of the patients' perspective, we also identified the GPs' view by describing their preliminary and work-up diagnoses after referral (table 5). In most cases, (n = 20, 29.0%), a multicausal aetiology (two or more possible causes) of dizziness had initially been assumed by the GPs. During the six-month follow-up period, n = 33 (47.8%) of the participants were referred to at least one specialist, including three patients that were sent to hospital because of symptom severity (suspicion of TIA, hypertensive crisis) or psychosocial reasons. In 6/33 cases (18.2%) the specialists' diagnoses were explicitly different from those of the GP, including two patients with newly diagnosed BPPV.

**Table 5:** GPs' preliminary diagnoses and referrals including specialists' (work-up) diagnoses.

Provisional diagnosis by GP	No. of patients (%)					
		Patients referred*				

			**Did not visit specialist**	**Confirmation of GPs' diagnoses**	**No attributable causes found**	**GPs' diagnoses if different from specialists' diagnoses**

multicausal†	20 (29.0)	10	0	1 BPPV 1 central	5	1 BPPV 1 orthostatic dysregulation 1 exclusion of presumed peripheral vestibular vertigo
cardiogenic	9 (13.0)	2	0	0	1	1 cervicogenic
cervicogenic	9 (13.0)	4	1	3	0	0
symptomatic‡	4 (5.8)	2	1	0	0	1 peripheral vestibular
peripheral vestibular	6 (8.7)	4	0	3	1	(in two cases specialists mentioned a central cause as additional differential diagnosis)
BPPV	6 (8.7)	2	2	0	0	0
vestibular neuritis	4 (5.8)	3	0	0	2	1 BPPV
psychogenic	2 (2.9)	0	-	-	-	-
Ménière's disease	1 (1.4)	1	0	1	0	0
central	1 (1.4)	1	0	0	1	0
none	7 (10.1)	4	3	0	1	0

Total n (%)	69 (100.0)	33/69 (47.8)	7/33 (21.2)	9/33 (27.3)	11/33 (33.3)	6/33 (18.2)

* Patients were referred to the following specialists (more than one referral per patient possible): otorhinolaryngologist (n = 20), neurologist (n = 10), orthopaedic surgeon (n = 7), cardiologist (n = 7), hospital (n = 3)

† at least two possible differential diagnoses

‡ e.g. during infection

## Discussion

### Summary of main findings

The present study demonstrates that a considerable aspect of older patients suffering from newly diagnosed, incident dizziness was associated with the dizziness handicap and reduced quality of life. However, mean scores of most outcomes (including dizziness handicap, quality of life, depression, activities of daily living) were only slightly affected and relatively stable during the six months of follow-up. This as well as the heterogeneous nature of the dizziness complaints in our sample made it difficult to clearly identify variables able to predict chronic dizziness. As to the patients' needs, knowing the cause of dizziness was repeatedly rated as very important. The empathy of the doctor was perceived as high, but this decreased over the study period. As to the GPs' management strategies, almost half of the patient study population was referred to a specialist. In two cases, benign paroxysmal positional vertigo (BPPV) was diagnosed only after specialist consultation.

### Strengths and limitations of the study

Whereas previous studies have mainly considered the overall outcome of dizziness [6,8,24], we prospectively assessed over time the intraindividual impairment of patients of a specific age group using the same set of validated instruments. However, it is necessary to mention that the sample size of our cohort was relatively small. This can be explained by closely framed inclusion criteria, which made recruitment of patients challenging. Obviously, incident dizziness was much less common in older patients than had previously been expected; however, the study had not been designed to allow identification of exact incidence rates. According to the participating GPs' experience, older patients did not usually indicate a starting point of their illness, but mentioned their symptoms only occasionally, if at all. For these reasons, we cannot be sure whether cases have really been included consecutively. In terms of diagnoses we relied -except in the referred cases- on the face validity of the GPs' diagnostic label. This was done in order to reduce the influence of the effects of a study protocol (Hawthorne effect) in favour of a description of the GPs' "usual" behaviour.

### Comparison with existing literature

In our study, chronically dizzy patients were more handicapped than temporarily dizzy patients; this was reflected by the fact that higher initial dizziness handicap scores (found from the questionnaires completed at the time of enrolment) tended to predict persistence of the symptom in our multivariate analyses. The majority of patients had a somewhat diminished quality of life in our study sample, however without a significant difference between temporarily and chronically affected patients. Other studies have shown similar or even more pronounced results in terms of handicap and a generally lower quality of life in dizzy patients [3,11,25], the latter being worse dependent on symptom duration. Similarly, Hsu et al. [11] and Tinetti et al. [13] found a correlation between symptom frequency and indicators of impaired functional and psychological outcome.

Former studies also revealed a high prevalence of psychological diagnoses [6,7] and a strong association between dizziness and psychological disorders such as depression and anxiety [3,7,12,13]. Similarly, in our study, self-reported anxiety was frequent initially. However, our patients (unlike those in other studies), did not suffer from depression at any time.

In general, comparability with other investigations was difficult: Most studies were cross-sectional rather than longitudinal, or patients were recruited in different settings (i.e. population-based). Another reason could be that a variety of assessment scales were used and, therefore, discrepancies were to be expected. As assessed by our own qualitative approach [26], the concept of dizziness handicap should contain both acute and a more general impairment. By considering both in the present study using the DiNA, handicap during acute episodes could be verified, which is an aspect that goes beyond the scope of the well-established DHI. The fact that the "importance of knowing the cause" was rated high and the perceived "empathy" decreased over time confirms findings of one of our previous studies [19] and might reflect a possible lack of patient-centeredness.

In terms of diagnostic strategies, a number of studies emphasised GPs' diagnoses based on medical history, clinical examination and follow-up as reliable and efficient [8,15,24,27]. However, Bird et al. [5] suggested further training of GPs was required because of inadequate referral for specialist consultation. Certainly, it is worth noting that in comparison to our data, Bird's study population was on average eight years younger, and the referral rate amounted to only 16%. The fact that 29% of our dizzy patients were not assigned a specific diagnosis is in accordance with other descriptions of a relatively high frequency of "unspecific dizziness" in primary care [28] or with understanding dizziness as a geriatric syndrome [4]. Our study confirmed BPPV as a relatively common [8], but frequently underestimated cause of dizziness among older people in primary care [29], although it can be easily assessed and effectively treated [30].

## Conclusions

Plausible factors indicating persistence of new-onset dizziness could not be identified, except an initially higher overall dizziness handicap. A patient-centred approach integrating functional impairments as well as patients' priorities, e.g. their need to understand causes, seems valuable in a situation where causative treatment is often not possible. This approach would also integrate psychosocial factors such as anxiety. Although dizziness is often understood as a multicausal geriatric syndrome in primary care, diagnostic algorithms would be useful and need to be evaluated so as not to miss treatable causes such as BPPV.

## Abbreviations

GP: General Practitioner; OR: Odds Ratio; CI: Confidence Interval; BPPV: Benign Paroxysmal Positional Vertigo; DHI: Dizziness Handicap Inventory; DiNA: Dizziness Needs Assessment; GDS: Geriatric Depression Scale; SF-12: 12-item Short Form Health Survey; ADL: Activities of Daily Living; iADL: Instrumental Activities of Daily Living; ICD: International Classification of Diseases; SPSS: Statistical Package for the Social Sciences; SD: Standard Deviation; TIA: Transient Ischaemic Attack;

## Competing interests

The authors declare that they have no competing interests.

## Authors' contributions

JS carried out the chart review, undertook the statistical analysis and interpretation of the data and drafted the manuscript. CK was responsible for the study concept and design, data analysis and interpretation and the preparation of the manuscript. BW participated in the statistical analyses and contributed to the preparation of the manuscript. EH-P developed the study concept and design, participated in the interpretation of the data and preparation of the manuscript. All authors read and approved the final manuscript.

## Pre-publication history

The pre-publication history for this paper can be accessed here:

http://www.biomedcentral.com/1471-2296/12/58/prepub
